# Small flexible automated system for monitoring *Caenorhabditis elegans* lifespan based on active vision and image processing techniques

**DOI:** 10.1038/s41598-021-91898-6

**Published:** 2021-06-10

**Authors:** Joan Carles Puchalt, Antonio-José Sánchez-Salmerón, Eugenio Ivorra, Silvia Llopis, Roberto Martínez, Patricia Martorell

**Affiliations:** 1 Instituto de Automática e Informática Industrial, Universitat Politècnica de València, Valencia, Spain; 2 Cell Biology Laboratory/ADM Nutrition/Biopolis SL/Archer Daniels Midland, Paterna, Valencia, Spain

**Keywords:** Electrical and electronic engineering, Imaging and sensing

## Abstract

Traditionally *Caenorhabditis elegans* lifespan assays are performed by manually inspecting nematodes with a dissection microscope, which involves daily counting of live/dead worms cultured in Petri plates for 21–25 days. This manual inspection requires the screening of hundreds of worms to ensure statistical robustness, and is therefore a time-consuming approach. In recent years, various automated artificial vision systems have been reported to increase the throughput, however they usually provide less accurate results than manual assays. The main problems identified when using these vision systems are the false positives and false negatives, which occur due to culture media changes, occluded zones, dirtiness or condensation of the Petri plates. In this work, we developed and described a new *C. elegans* monitoring machine, SiViS, which consists of a flexible and compact platform design to analyse *C. elegans* cultures using the standard Petri plates seeded with *E. coli*. Our system uses an active vision illumination technique and different image-processing pipelines for motion detection, both previously reported, providing a fully automated image processing pipeline. In addition, this study validated both these methods and the feasibility of the SiViS machine for lifespan experiments by comparing them with manual lifespan assays. Results demonstrated that the automated system yields consistent replicates (p-value log rank test 0.699), and there are no significant differences between automated system assays and traditionally manual assays (p-value 0.637). Finally, although we have focused on the use of SiViS in longevity assays, the system configuration is flexible and can, thus, be adapted to other *C. elegans* studies such as toxicity, mobility and behaviour.

## Introduction

*C. elegans* has been successfully used as a biological model for a wide range of studies related with development, longevity and diseases. In particular, lifespan assays in *C. elegans* has become one of the most widespread research trial models.

In biological terms, *C. elegans* has a lifespan of approximately 3 weeks and it is sensitive to environmental conditions, such as light and temperature. Thus, phototaxis^1^ and thermotaxis^2^ stimuli should be considered when performing lifespan assays as they could impact on the nematode’s life expectancy in extreme conditions. In *C. elegans*, phototaxis is characterized by a withdrawal response from wavelength light source shorter than blue light^3^. Thus, the lower the wavelength, the greater the nematode’s response. Light not only causes a simple escape response, but also causes death when blue-violet and at shorter wavelengths^4^. In the case of thermotaxis, nematodes also exert a withdrawal response to a heat source^5^. Just slight heat exposure induces hyperthermia, which reduces life expectancy and causes death. Both stimuli cause stress in the nematodes, therefore, protocols are based on storing cultures in temperature-controlled incubators under dark conditions during the assay, exposing them to ambient conditions only in short inspection periods (a few minutes every day) under the dissection microscopes.

Traditionally the *C. elegans* lifespan assay^1, 6^ has been performed manually by expert inspection using a dissection microscope, and counting every day the number of nematodes surviving under specific culture conditions^7–11^ in standard Petri dishes.

The dead or alive criterion is inferred from C. elegans movement, which categorizes the worm as alive if movement is detected, and dead otherwise. However, this is complicated due to animal slowness in accordance with its ageing, which ends when worms fail to move at all during inspections. This is why nematodes are mechanically stimulated by an expert applying pick stimulation to confirm death. This task, which has to be done to each worm successively, is both arduous and laborious.

The difficulty arises when assays require large numbers of nematodes. In this context, there is a need to increase the screening capacity, reduce errors and produce objective results.

Different automated lifespan systems have been described for studying *C. elegans* viability, mobility or fecundity. Most of them capture and process images of *C. elegans* cultured in different constrained scenarios to facilitate detection of the time of death. Some systems use standard Petri dishes, such as Lifespan Machine (LM) and SiViS. On the other hand, there are systems, such as WorMotel, microfluidic devices and others, that use non-standard plates.

The Lifespan Machine (LM), is an example of a medium-scale-plate image-capture device for macro-inspection ($8\,\upmu$m), and is based on the simultaneous capture of 16 standard Petri plates per scanner^12^. Although it is based on automated detection, this device only allows a small number of frames to be monitored per minute due to the slow image scanning process. Besides, a large number of plates in an assay will require many scanners, which may make the conditions heterogeneous, for the same measurement. In addition, the plates cannot be exchanged, and remain fixed in the equipment until the end of the experiment, thus preventing the devices from being used in parallel for other assays. In order to increase flexibility, our device has managed to take enough images to track the *C. elegans* paths. This feature also enables it to perform other kinds of assays (such as dispersion, healthspan, etc.). In addition, it allows the Petri plates to be changed during the experiments, as well as measuring plates under the same conditions.

Another example of a lifespan-assay machine is the WorMotel^13^. This machine automates the plate-changing process, allowing for large-scale screening under the same conditions by using a robot handling system, which transports plates from buffers to inspection zones. However, it uses non-standard plates, where each well contains only one *C. elegans* to simplify death detection. It should be pointed out that worm behaviour might be modified due to isolation in a small space^14^, and this set up does not allow long-trajectory analysis. By contrast, in our system, worms are inspected in standard Petri plates measuring 55 mm in diameter, holding 10-15 individuals and thus permitting more complex social behaviour^15, 16^.

Further examples of inspection equipment are based on microfluidics with techniques such as micropillars^17–19^, which consist of keeping the nematodes between micropillars. The advantage is that fluid can be replaced by keeping the worm in position; however, it is subject to mechanical stress. These machines use non-standard plates and are therefore often difficult to adopt by traditional laboratories, where standard Petri dishes have been used for many years.

There is also open-source software to process image sequences, either to detect the nematodes or even to extract subtler characteristics that may provide other types of information. These include trackers that observe an individual worm^20^, multi-trackers that follow the progress of several individuals^21^ and microscopy devices that extract micro-features from a worm^22^. There are also several tools for animal detection and phenotype identification. Phenotypes for motility^21, 23–26^, longevity^27, 28^ or fecundity^29^, among others.

The goal of our study was to validate the methodologies and a new automated system for *C. elegans* longevity studies. Our system is characterized by its compact, flexible, scalable and used-friendly design. This newly described method for *C. elegans* lifespan assays is based on the integration of different computer vision techniques, such as active vision^30^ and adaptive motion detection techniques^31^, which we evaluated together. The active vision method improves image quality and minimises the light intensity emitted by the lighting system illuminating the worms. The other method, motion detection, also implements a post-processing adaptive data filter; this method improves worm detection. All in all, this system enables the automation of *C. elegans* lifespan assays using computer vision techniques in a scenario similar to the manual assays. The new method described herein draws on the traditional manual *C. elegans* lifespan inspection method, but incorporates fully automated image processing methods. This paper begins by describing the SiViS machine and methods, which include building a prototype, and the computer vision techniques employed. Secondly, we report lifespan assays performed to compare the automated SiViS results with manual expert’s results for validation proposes. Finally, we draw conclusions and suggest future studies.

## Materials and methods

To test the proposed automated method, a SiViS prototype has been designed, developed and validated. Thus, several methods are explained in the next subsections: (1) the SiViS and software GUI designed and (2) the light control, image processing and lifespan validation employed.

### Compact mechanical design

**Figure 1:**
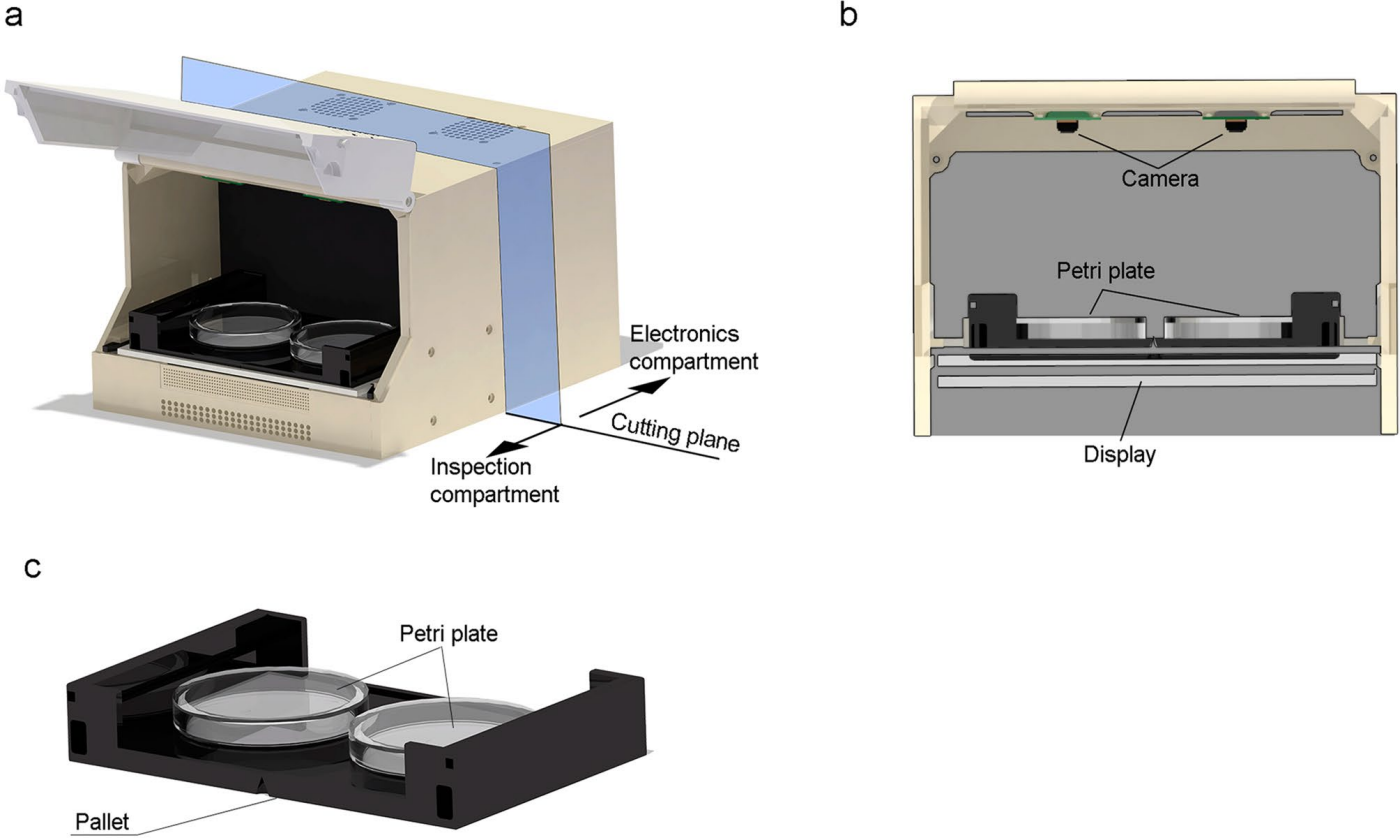
Prototype of the SiViS device. The image was obtained with SOLIDWORKS 2020 SP3.0. (**a**) SiViS with the open door, showing the pallet inside the inspection area. The electronic compartment, at the back, is closed and therefore hidden from view. (**b**) Section showing the inspection area and the distribution of the components. (**c**) Pallet with two slots for the Petri plates.

SiViS has two closed compartments: one for the inspection area (Fig. 1a, b) and the other for electronics. The closed inspection compartment attenuates the environmental conditions of lighting and temperature, to minimise them and thus reduce their direct effect on nematode life expectancy. There is a door for easy introduction and replacement of Petri plates. Forced ventilation ensures that the inspection area stays at room temperature (Supplementary Figure S1) to prevent condensation and hyperthermia, and a sensor registers the temperature and inserts it as metadata in each image. The inspection area has a slot where the pallet (Fig. 1c) is fitted.

The Petri-plates fit into the pallet (Fig. 1c) facilitating storage, as well as improving their handling and correct placement in the inspection area. The design was conceived to include two slots for two Petri plates. The pallet is rectangular ($140\times 90$ mm), with two circular holes inside measuring 54 mm in diameter, into which the two 55mm-diameter Petri plates are slotted. It is rigid with just a small amount of flexible material for the circular plate-slots, allowing the necessary deformation for the plates to slot in. The pallet has two grooves by which it can be fitted into the device frame, thus restricting potential shifting and rotation. The pallet rests on the lighting system diffuser. All of these features avoid displacement and ensure that the plate is always in a similar position, in order to improve repeatability.

The vision system in the inspection compartment is configured in a traditional backlight way, with the lighting system below, Petri plate above, and camera overhead (Fig. 1b). This device has two 5MP cameras to inspect each Petri plate, thus enabling the parallel inspection of two Petri plates of 55 mm diameter.

### Prototype construction, components and assembly

The mechanical components were produced by 3D printing, with the fused filament fabrication (FFF) method. Materials employed were polylactic acid or polylactide (PLA), which melt at between 150 and $160^{\circ }$C. There is an elastic piece to forcibly fit the plate onto the pallet, this material is Filaflex, and was fabricated in the same process.

Electronic components: 7” Raspberry Pi display of $800\times 480$ pixels size, $155\times 86$ mm visible area and 16.7 million colours, Raspberry Pi v3, Raspberry Pi NoIR Camera v1, Multiplexor for Raspberry Pi Camera. The components and the guidelines to build this system and the assembly description can be found in the repository https://github.com/JCPuchalt/SiViS.

### Lighting control

**Figure 2:**
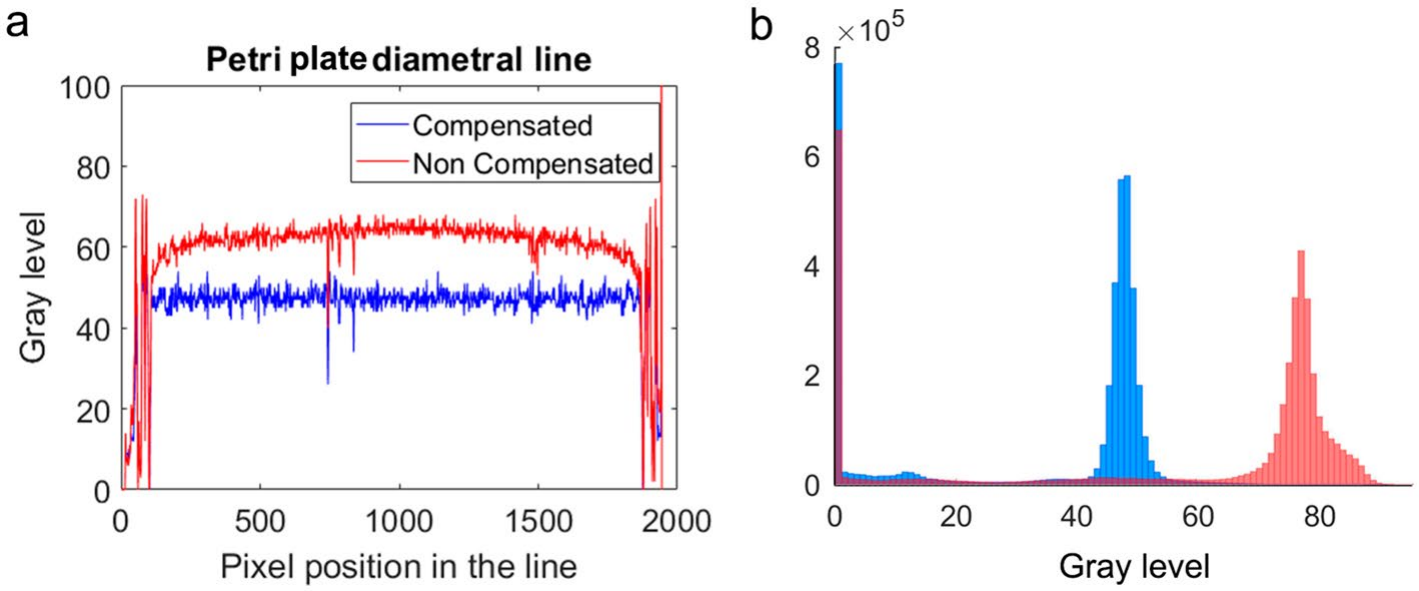
Light control results. The image was obtained with MATLAB R2020b. (**a**) Two images were taken of the same plate, one image was not compensated (red) while the other was compensated (blue). For comparison, the grey pixel level is shown in diametral line (profile line) to the Petri plate of each image. The diametral line measurements correspond to the intensity line profile. Compensated image tends to reference level (48). (**b**) The compensated image histogram shows the background level approximates the reference level (48).

To help automate image segmentation we used the method described in a previous work^30^, which is based on keeping the image pixel level at a reference value as shown in Fig. 2b, by regulating light intensity dot to dot from the lighting system. This system uses the display as a lighting system (actuator) onto which a different projection pattern is drawn at each sampling time. The camera also functions as a sensor, feeding-back the signal and adjusting the control action to obtain a constant reference throughout the entire image. This method enhances worm definition and improves image visibility despite certain kinds of dirtiness and condensation.

A proportional controller (PID) was designed to cancel out errors in few iterations. Errors are the differences between references and measurements. The references are the desired intensity levels in the images. The measurements are the intensity levels in the captured images. The control actions, calculated by the regulator, are the light intensities of the light points. An automated calibration process was performed to obtain the homography relation between pixels and points of light.

*Caenorhabditis elegans* are sensitive to blue light^13, 32^, so an important aspect of this system is that we can select the amount of blue, green and red that the nematode is subjected to, and thus control the levels of light stress imposed. To regulate light intensity, we used the strategy of increasing the RGB components of each pixel by first increasing the red component progressively. When red reaches saturation, green is added progressively to the saturated red (255), after green becomes saturated too, the same is done with blue component. The parameters were configured so that the blue component was not required for control action compensation, however in the opaque zones the blue component was required to try to compensate. These dark areas are the plate ends, since the plate walls generate shadows (Fig. 2a).

### Flexible image processing techniques

These techniques^31^ are based on nematode *motion detection* and *post-process* filter. During this assay, several *C. elegans* were cultured in standard Petri plates, which were transported daily from the incubator to the inspection zone.

The active lighting system allows a fixed threshold segmentation algorithm to automatically extract dark blobs in all images captured with SiViS. After some image processing steps, a blob filter is applied. This filter is based on features of shape, color and movement. Finally, live worms are detected based on movement features extracted from different days.

As previously stated, the pallet was designed to improve repeatability for plate replacement, and software^31^ was employed to compare nematode position on different days. This software estimates the translation and rotation of nematodes and transforms this data to match blobs between images.

#### Motion detection techniques

Nematode movement slows down with ageing, so this is an important feature to observe when tracking worms. Likewise, data on maximum velocity must be established. After observing 10 plates and taking into account numerous studies^20, 21, 33^, worm speed was estimated at between 0.2 mm/s and 0.5 mm/s, which represents 50% of its size per second. Thus, taking an image per second ensures overlap of the nematode in each consecutive image, which makes tracking easier and more accurate. Regarding *nematode motion*, at least a sequence of In images per plate ($I_n=30$) were taken every day at 1 fps. The image resolution achieved was $30\,\upmu$m/pixel, which provides enough information for behavioural analysis, and also captures the entire plate. Movement integration was extracted from this sequence (Supplementary Figure S2). This motion information can be observed in this sequence when worms are young; however when they age their movement has to be compared with the sequence taken on the previous day. If nematode position does not change from one day to the next, then the worm is dead.

Lifespan automation is challenging because a host of problems can arise. The image processing software must be designed to avoid different causes of false-negatives (or undetected live worms) and false-positives (or wrongly detected live worms). False-negatives can be due to worm aggregation problems or to occluded plate zones (e.g. zones near plate walls, or non-transparent zones due to dirt or condensation problems). False-positives can be due mainly to dirt problems.

#### Post-process filter technique

Lifespan curves are monotonically decreasing functions. Therefore, a lifespan-counting error could be detected when a current live worm count was higher than a previous count. These errors can occur for two different reasons: (1) because the live worms detected in the current image sequence were aggregated or hidden in previous sequences (previous false-negatives) or (2) because some blobs, which erroneously appeared due to dirt (dust spots on lids), met the live worm criterion in the current sequence (false-positives).

A post-processing method is proposed in an attempt to optimally correct these errors. It is noteworthy that corrections were made only if a count error was detected in the automatically extracted lifespan curves. Corrective actions took into account error occurrence probabilities in order to act accordingly.

In the first stage (half of the lifespan cycle), more potential errors appeared due to hidden worms and aggregation (false-negatives) than to dirt (false-positives), and survival was high (Supplementary Figure S2). In the second stage (half of the lifespan cycle), this situation was inverted (dirt accumulated and survival dropped). Consequently, the post-process contemplated these two stages. In the first stage, if an error was detected the previous count (Supplementary Figure S3) is corrected upwardly and in the second stage actual count is corrected downwardly.

This post-process method corrects the errors detected taking into account the different probabilities of occurrence of the errors in two different phases separated by a threshold. This threshold can be defined as the meanlife of the strain used in the assay.

### User-friendly software

**Figure 3:**
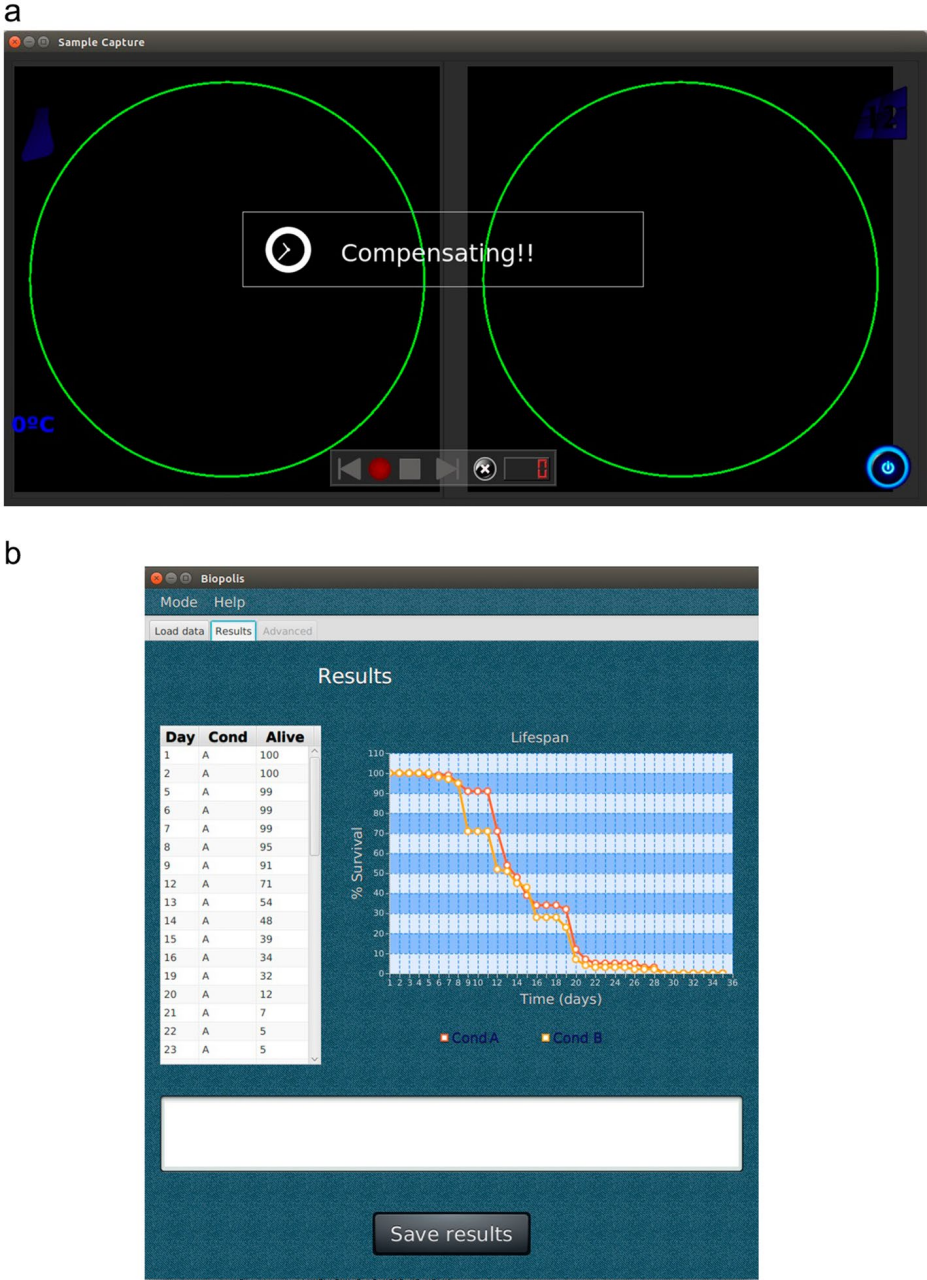
Graphic user interfaces. (**a**) CaptureCE interface is like a video player which shows the two pallet plates in real time. Experiments are created and the experiment to be captured is selected. (**b**) ProcessorCE interface is used to select captured experiments to be processed and provide the corresponding survival results.

In order to manage the daily image-capture tasks, two software applications were created on a server (desktop PC) (Fig. 3). One is the CaptureCE software, which enables the user to design experiments easily in a graphical interface, automatically programming image-acquisition tasks in a user-friendly way. Thus, the user only has to click on a button to take the images, and see them in real-time; consequently, if contamination is shown, the contaminated plates can be censored. The images are sent from SiViS to server via Ethernet. The other software is the ProcessorCE (Fig. 3b), which enables image processing and survival-curve creation by clicking on a button, as easily as with CaptureCE (Fig. 3a).

The use of this device is simple. Petri plates are fitted into pallets, which are stored inside an incubator. Pallets are later removed from the incubator and fitted into the SiViS slot. Once pallet images have been taken, the pallet in the SiViS slot is exchanged for another one for inspection, and so on. Pallets are numbered, in order to follow a sequential procedure, whose sequence matches the programmed sequence of tasks automatically created by the CaptureCE software. Hence, the user follows the procedure, which noticeably reduces human errors in this process.

### Lifespan validation

#### *Caenorhabditis elegans* strains and culture conditions

*Caenorhabditis elegans* strains N2, Bristol (wild-type); CF1038, *daf-16 (mu86)* and CB1370, *daf-2 (e1370)* were obtained from the *Caenorhabditis* Genetics Center at the University of Minnesota. All strains were maintained at $20^{\circ }$C on nematode growth medium (NGM) seeded with *Escherichia coli* strain OP50, as standard diet.

#### Lifespan assays

Lifespan assays were performed with the wild-type strain N2 or the corresponding mutant strains deficient in *daf-16* (CF1038 (*daf-16, mu86*)) and *daf-2* (CB1370, *daf-2 (e1370)*. Age-synchronized worms were obtained by hatching the eggs from gravid worms in NGM plates and incubating them at $20^{\circ }$C until the young adult stage was reached. FUdR (0.2 mM) was used to prevent reproduction^34, 35^, and fungizone ($1\,\upmu$g/mL) was added to prevent fungal contaminations. The drug metformin was added to NGM at a dose of 50 mM. Young adult worms (10-15 per plate) were used for the first day of lifespan experiments. Viability was scored daily (except weekends) with the automated SiViS device until 100% of nematodes were dead. In parallel, manual experiments were performed. In these experiments, nematodes were scored under a dissection microscope and considered as dead if they failed to respond on being prodded with a platinum wire. At least, two independent assays were carried out in each test.

#### Statistical analysis

Statistical significance among lifespan curves was estimated by log rank test using GraphPad Prism v. 4 statistical software. This test was used to analyse reproducibility on *C. elegans* N2 lifespan curves obtained in the automated SiViS system. Furthermore, correlation between manual and automated experiments was evaluated by comparing lifespan curves in N2 strains and the mutant strains in *daf-2* and *daf-16*, exhibiting extended or shortened lifespan.

## Results and experiments

SiViS integrates different techniques, which might lead to some unexpected behaviour. In order to assess the correct functioning of the system, several experiments were performed. Thus, lifespan curves data from different trials were used to asses various features, such as repeatability and correlation between manual and automated count. The correlation was performed for wild-type strain N2 strain and for mutants CF1038, *daf-16 (mu86)* (short-lived strain) and CB1370, *daf-2 (e1370)* (long-lived strain). In addition, the anti-diabetic drug metformin, which has been reported to increase *C. elegans* lifespan, was included in the validation study^36–38^.

### Automated system provides repeatable results

Lifespan measures, in particular, are well known to exhibit substantial run-to-run variability in *C. elegans*, due to stochastic events^39^.

**Figure 4:**
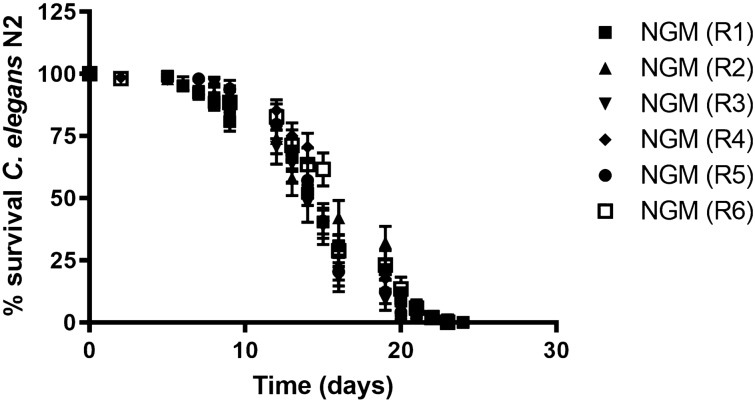
N2 survival curves. The graph was obtained with GraphPad Prism v. 4. Lifespan curves of *C. elegans* N2 obtained from independent experiments with the automated system, in standard conditions (NGM). Curve comparison was estimated by Log RanK T-test (*p* value: 0.699). A comparison among replicates is indicated in Table 2.

In order to determine the repeatability of the method for counting live nematodes in the automated device, we obtained different lifespan curves from six independent experiments (Table 1) with wild-type nematodes in standard growth conditions (NGM plates). Results, shown in Fig. 4, indicate low levels of deviation among the different curves measured in the device. We obtained overlapping curves, showing little variation in median lifespan (p-value log rank test: 0.699). Therefore, we can conclude that the automated system produces consistent replicates (Table 2). This further demonstrates the high degree of homogeneity and repeatability offered by this system.

**Table 1:** Sample size and number of plates are indicated for each independent assay with wild-type *C. elegans* strain N2 in standard growth conditions (NGM plates).

NGM replicates	Sample size	Nr. plates
R1	104	8
R2	50	4
R3	44	4
R4	68	6
R5	49	4
R6	52	4

**Table 2:** p-values estimated in the comparison among replicates of NGM.

P value					
NGM replicates	R2	R3	R4	R5	R6
R1	0.4796 (ns)	0.6951 (ns)	0.3124 (ns)	0.5324 (ns)	0.37775 (ns)
R2	–	0.2043 (ns)	0.8660 (ns)	0.1210 (ns)	0.9928 (ns)
R3	–	–	0.1750 (ns)	0.9821 (ns)	0.3029 (ns)
R4	–	–	–	0.668 (ns)	0.9621 (ns)
R5	–	–	–	–	0.1296 (ns)

A Log Rank t-test was applied.

ns: without significant differences

### Lifespan curves of wild-type *C. elegans* obtained in the automated device correlate with manual assays

A critical test of the automated system developed is to verify that nematode lifespan measured by this device is similar to that for animals reared under conventional agar-plate-based conditions. To do so, we performed parallel studies of animals maintained in NGM plates, and recorded live worm-counts by machine and using specialized technicians.

**Figure 5:**
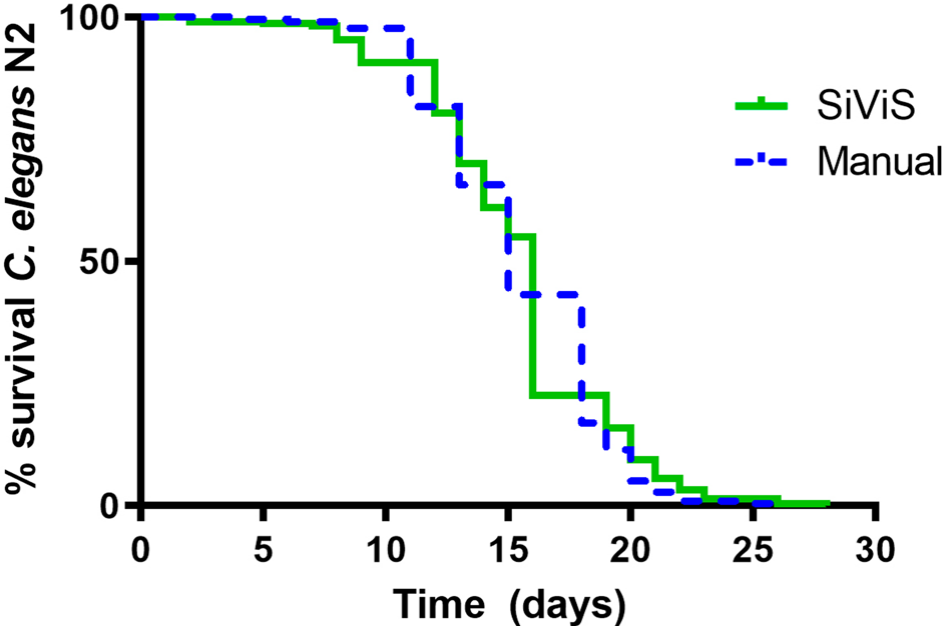
Manual-automated comparison for N2. The graph was obtained with GraphPad Prism v. 4. Comparison of lifespan curves obtained by the automated system and by a manual assay for *C. elegans* wild-type strain N2. Data from four independent experiments ($n=200$/condition; 16 plates/condition). To compare curves a Log Rank T-test was applied (*p* value: 0.637). Mean lifespan of 16 days (for automated system) and 15 days (for manual assay). Maximum lifespan of 26 days (automated and manual assays).

Survival curves obtained for *C. elegans* N2 by the automated system were statistically similar to those obtained by manual worm-count assays. As shown in Fig. 5, very similar results were obtained for both approaches, without significant differences between both curves (p-value: 0.637).

### Validation in *C. elegans* mutant strains

The IIS pathway is the best-characterized longevity pathway in *C. elegans*. Reduced signalling through this pathway leads to the activation of FOXO-family transcription factor *daf-16*^40, 41^ and results in robust lifespan extension. Reduction-of-function mutations or RNAi knockdown of several components of this pathway, including the genes encoding the insulin-like receptor *daf-2* or the PI3-kinase *age-1*^42^, have been reported to enhance longevity.

Accordingly, our next step was to analyse whether mutations known to modify lifespan also produced the expected phenotypes in our automated system. Lifespan curves from mutant strains for CF1038, *daf-16 (mu86)* and the long-lived CB1370, *daf-2 (e1370)* were obtained both with SIVIS and manual assays.

**Figure 6:**
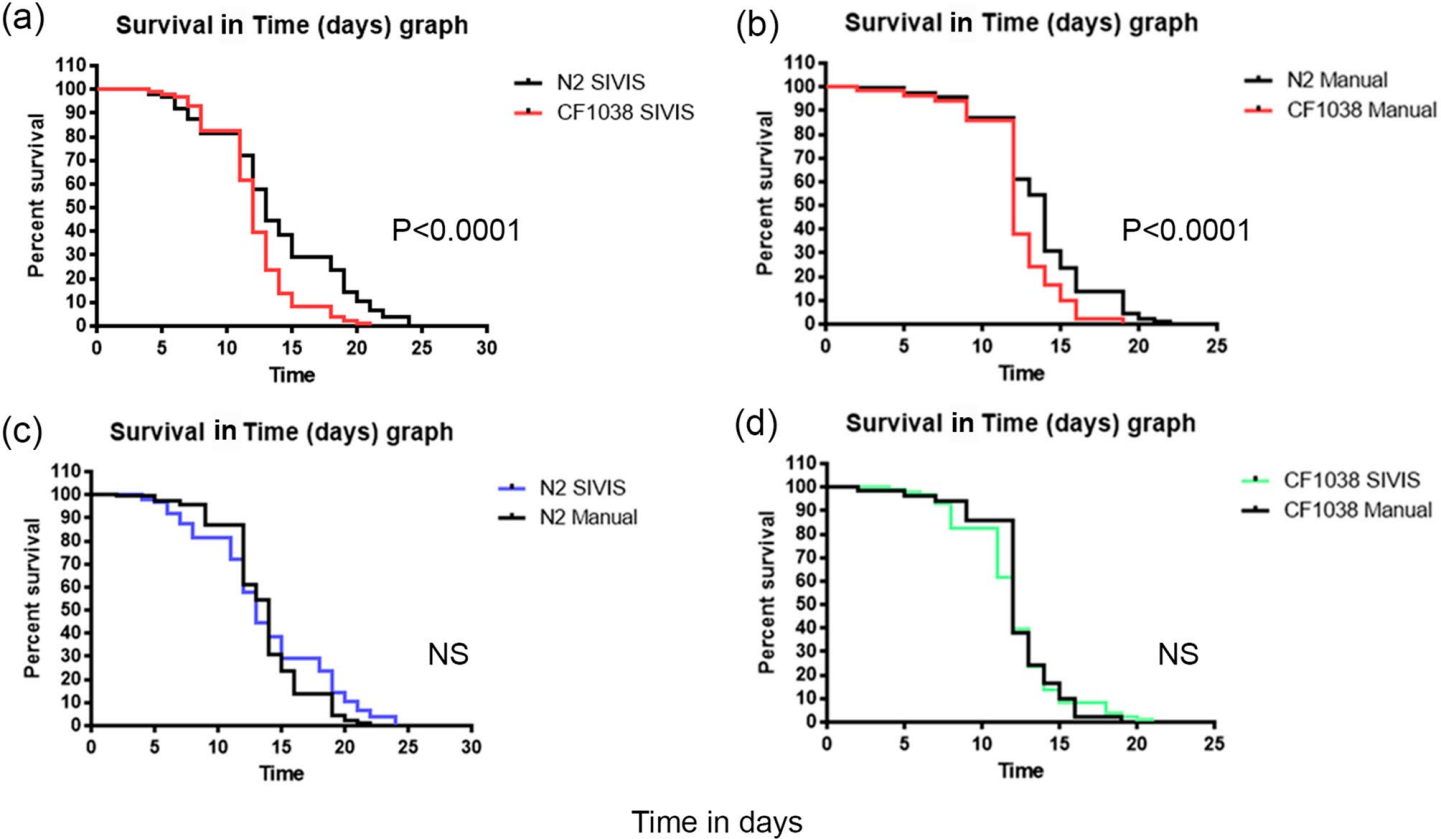
Lifespan of short-lived strain CF1038, *daf-16 (mu86)*. The graph was obtained with GraphPad Prism v. 4. (**a**) and (**b**) show the comparison between survival curves for CF1038 and the wild-type strain N2 in assays performed in SIVIS and manually, respectively. (**c**) and (**d**) show the comparison between the manual and automated system SIVIS for each strain. Data from three independent assays for automated assays ($n=300$ and 24 plates/condition) and two for manual assays ($n=200$ and 16 plates/condition).

As shown in Fig. 6, the mutant strain for *daf-16* exhibited a significantly shortened lifespan compared with the wild-type strain N2 ($P<0.0001$), both for manual and automated results (Fig. 6a, b). Thus, a mean lifespan of 13 days (for N2) and 12 days (for CF1038) was obtained in the assays scored in SIVIS, and 14 days (for N2) and 12 days (for CF1038) in the manual assays. These results indicate that the automated system significantly detects a shortened lifespan in the case of CF1038, *daf-16 (mu86)*, as reported in the literature. Furthermore, the comparison between the manual and automated systems for each strain clearly shows very similar survival curves (Fig. 6c, d).

**Figure 7:**
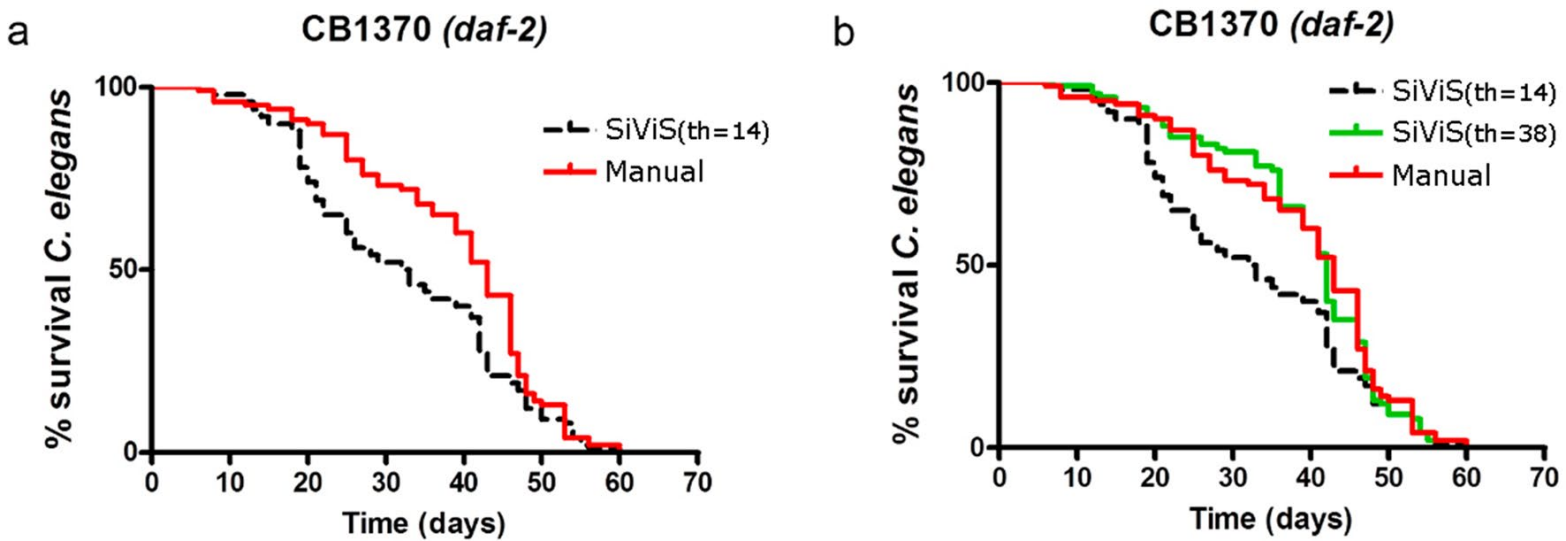
Manual and post-process lifespan comparison of *daf-2* mutant strain. The graph was obtained with GraphPad Prism v. 4. (**a**) Curves obtained in SIVIS differed significantly using the standard correction algorithms with the threshold (th) set in 14 ($th=14$). (**b**) Post process of correction algorithms set in $th=38$ enabled correct survival measurement of lifespan in long-lived strains like CB1370 (*daf-2*). Data from two independent assays ($n=100$ and 8 plates/condition).

This correlation was also observed for the long-lived strain *daf-2*, but it was necessary to modify the threshold of the post-process filter method (stated in the Flexible image processing techniques subsection). For the common lifespan strains the threshold was about the meanlife (14th day), nevertheless for this longer lifespan strain the threshold should be later (Supplementary Figure S4). As shown in Fig. 7, curves without this correction differed from the manual assay, while a post-process correction of algorithms enabled a correct survival measurement for lifespan in the long-lived strain CB1370 (*daf-2*).

### Validation of metformin

Metformin is a widely used first-line drug for treatment of type 2 diabetes and has been shown to extend lifespan. Recently, it was shown that metformin increases *C. elegans* lifespan via the lysosomal pathway^36, 43^.

**Figure 8:**
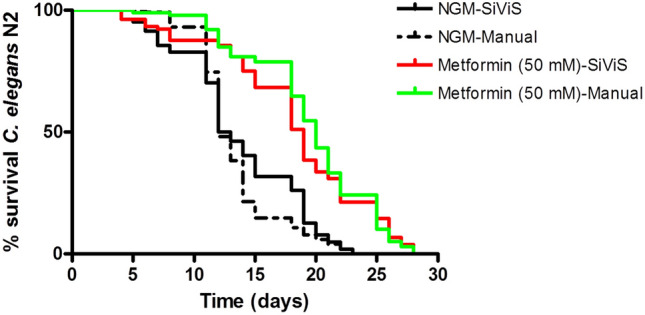
Manual-automated comparison for Metformin. The graph was obtained with GraphPad Prism v. 4. Effect of metformin on *C. elegans* lifespan measured in manual lifespan assays and in the automated SIVIS system. Data from two independent assays ($n=100$ and 8 plates/condition).

In this study, metformin was used at 50 mM, as a dose that previously showed positive effects on *C. elegans* longevity^37^. Manual lifespan experiments were performed in parallel with experiments in the automated system. Results indicated that, as previously described, metformin significantly increases *C. elegans* lifespan, both in the manual ($P<0.0001$) and in the automated system ($P<0.0001$) (Fig. 8). Furthermore, the mean lifespan in the presence of metformin increased 66.6% (manual assay: $NGM=Day 12,Met=Day 20$) and 52% (automated assay: $NGM=Day 12.5, Met=Day 19$). No significant differences were observed between manual and automated curves for the different feeding conditions (p-value control:0.1820; p-value metformin: 0.469).

## Conclusions

Here we have presented a new automated method that reproduces the results of the traditional *C. elegans* lifespan method. This automated method frees the expert from the repetitive and arduous work involved in manually counting large nematode populations. It is also a flexible system because, in addition to lifespan assays, it can be used to measure other *C. elegans* phenotypes, such as those related to dispersion, healthspan, memory or food preferences, among others, by simple code modification. Moreover, it is small and use-friendly, enabling more than one experiment to be conducted in parallel on the same device, since the plates can be removed from the inspection area even though the experiment is still underway.

This new method using the SiViS monitor is based on two techniques: i) active vision^30^ and ii) flexible motion detection^31^. This live/dead criterion facilitates accurate daily worm-counts, even though the plate is removed and replaced each day. By being able to change the plates, several experiments can be monitored at the same time, which provides greater productivity and throughput. This also makes it more flexible, so the experimental conditions can be customized. The other technique proposed here relates to active vision, which is an area of computer vision, achieved by adding a control system. Intelligent illumination may highlight the image characteristics of interest, making the light intensity more uniform. This facilitates image segmentation by providing more refined images, which could reduce processing time. Intelligent illumination is a controlled illumination system, which regulates the light intensity and components to which the worm is subjected and, therefore, a lighting pattern can be defined in order to stress the nematode as little as possible, while maintaining optimum image quality.

In this work, a SiViS prototype has been designed, developed and validated for lifespan assays. Results show it is a good approach for lifespan assays in automated worm-count using the methods described herein, without significantly statistical differences between automated and manual counts. Moreover, no significant differences were found between independent measurements, thus its repeatability is good. These conclusions are not only for the N2 strain, but also apply to metformin treatment or to other strains (*daf-2* and *daf-16*), whose lifespans are up to two or three times longer, or shorter. Therefore, the methods are suitable for a variety of lifespan assays.

Considering that SiViS is a satisfactory measuring system, given the above, it has several other advantages. The first relates to the possibility of replacing the Petri plates during the experiment, thus enabling the monitoring of new experiments at any moment. Another advantage is its small size, thus it can be easily scaled up by increasing device number. In addition to this, plate replacement could be automated to achieve a fully autonomous lifespan assay process.

In a future work, we will endeavour to increase sensitivity, which should reduce variance error. If variance error were reduced, sample size might also be reduced, which would save time, number of animals and storage space. In order to achieve this improvement in sensitivity, one solution might be to stimulate worms to make them move, making it easier to detect them. Several other options exist to create this stimulus, such as phototaxis, chemotaxis or vibrotaxis.

## Supplementary information

Supplementary Information.
