# Supplementary material for: Small flexible automated system for monitoring Caenorhabditis elegans lifespan based on active vision and image processing techniques
Source: Sci Rep. 2021 Jun 10;11:12289. doi: 10.1038/s41598-021-91898-6 (PMC8192789; doi:10.1038/s41598-021-91898-6)
Supplement: Supplementary file 1 — Supplementary Information. [file 41598_2021_91898_MOESM1_ESM.pdf]

Small flexible automated system for monitoring *Caenorhabditis elegans* lifespan based on active vision and image processing techniques

Joan Carles Puchalt[1], Antonio-José Sánchez-Salmerón[1,*], Eugenio Ivorra[1], Silvia Llopis[2], Roberto Martínez[2], and Patricia Martorell[2]

[1]Universitat Politècnica de Valéncia, Instituto de Automática e Informática Industrial, Valencia, Spain
[2]Cell Biology Laboratory/ADM Nutrition/Biopolis SL/Archer Daniels Midland, Paterna, Valencia, Spain
[*]asanchez@isa.upv.es

Contents

Temperature profiles 3
Image processing results in a usual scenario 4
Image processing results in a dirt scenario 5
Manual and automated lifespan comparison of daf-2 mutant strain. 6

Temperature profiles

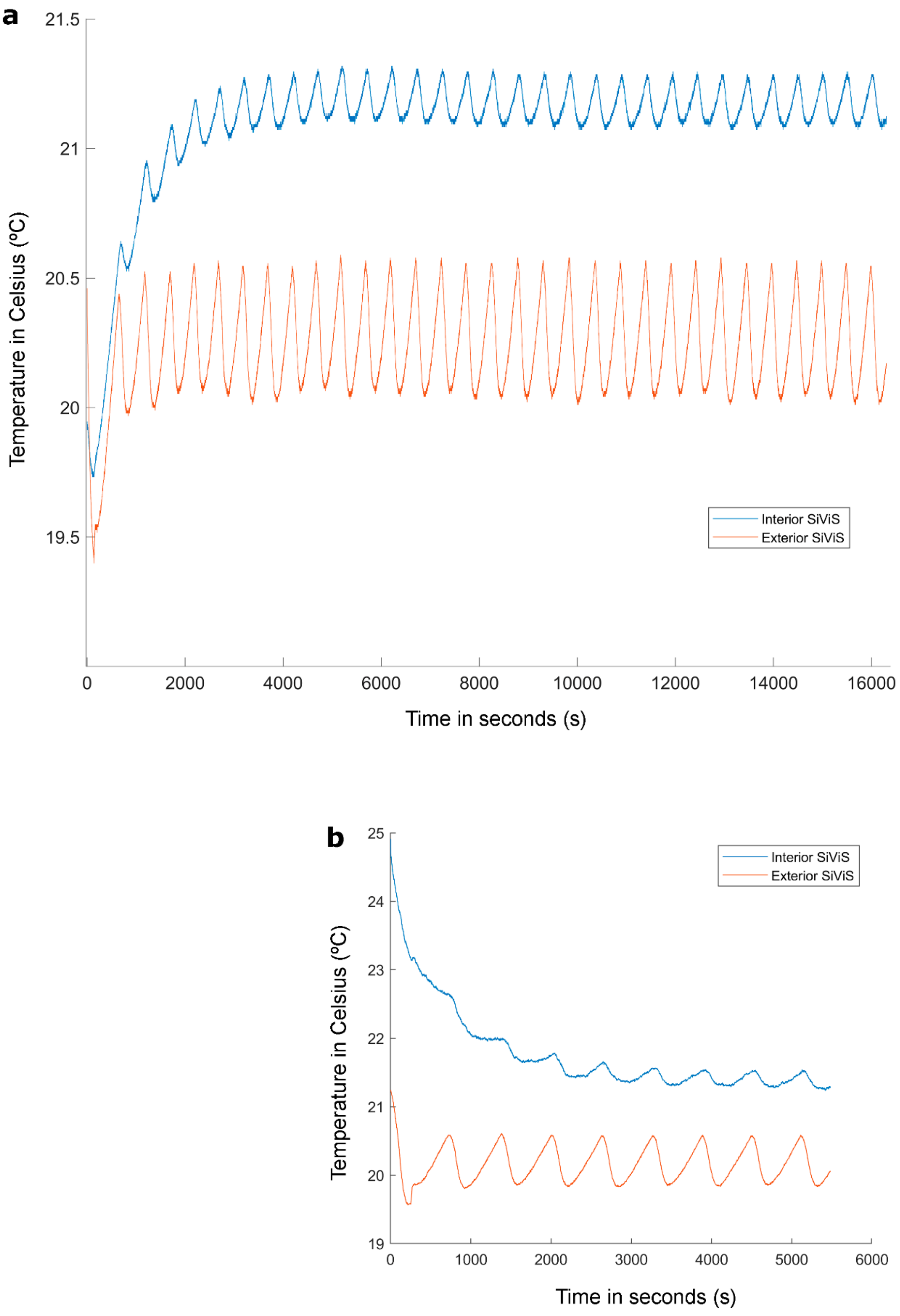

Supplementary Figure S1. Temperature profiles. The graph obtained with Matlab R2020b. The temperature is taken outside (red curve) and inside (blue curve) of SiViS. SiViS is inside a room at approximately 20ºC. a) shows the temperature recorded for 16000 seconds (more than 4 hours). Throughout this measurement, the SiViS interior temperature never exceeds one degree of difference with respect to the SiViS exterior. b) shows the same behavior, but having introduced SiViS into the room a few minutes before conducting the experiment, so the device is initially at approximately 25ºC, and little by little it drops to the room inside temperature. It

can be shown that the SiViS design allows that during its operation it only increases +1ºC with respect the external temperature.

Image processing results in a usual scenario

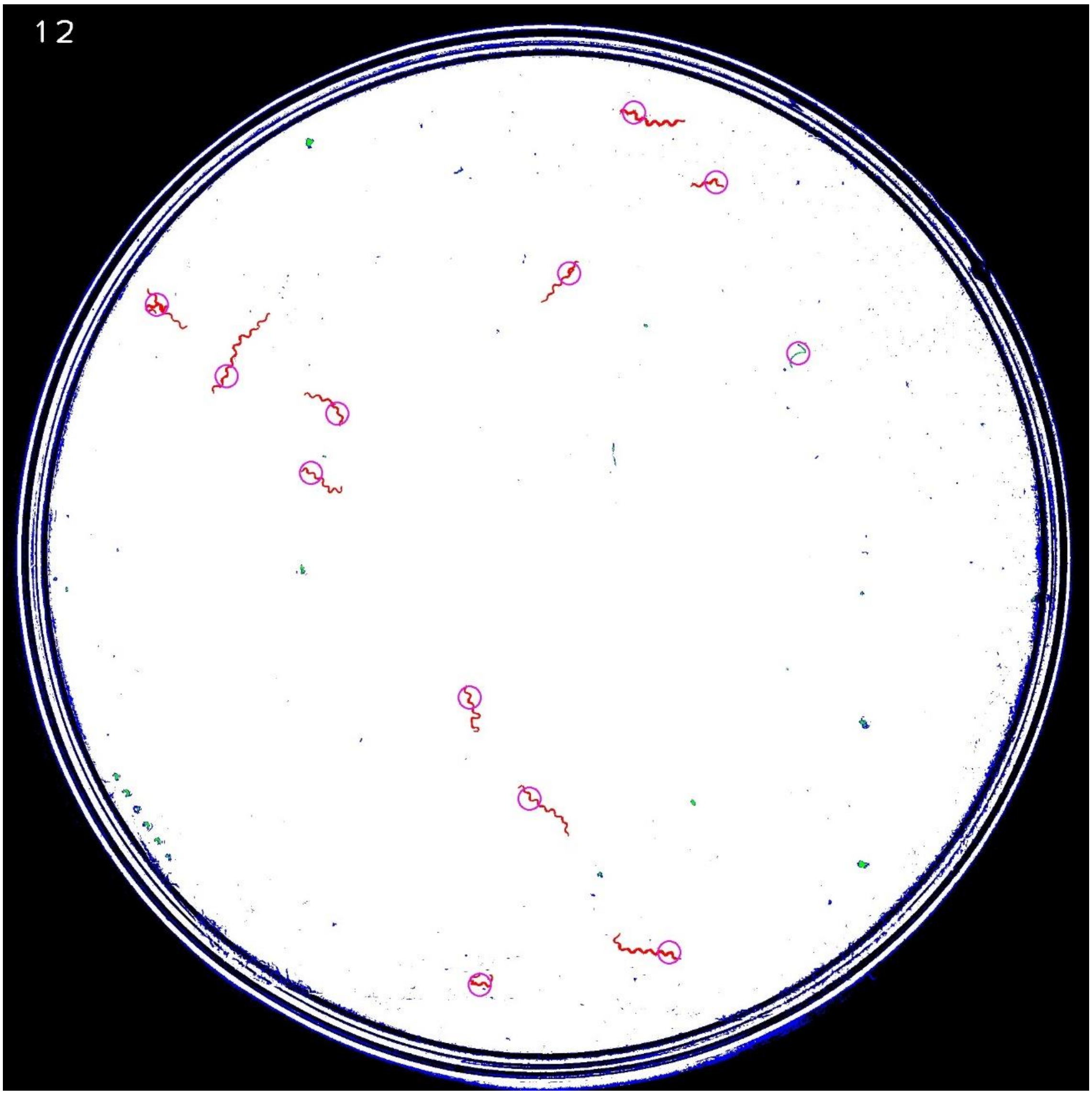

Supplementary Figure S2. Image processing results in a usual scenario. In this case, twelve live worms were detected. Each track (or movement integration) during the image sequence is shown in red. Each living worm is shown in the last image into a pink circumference.

Image processing results in a dirt scenario

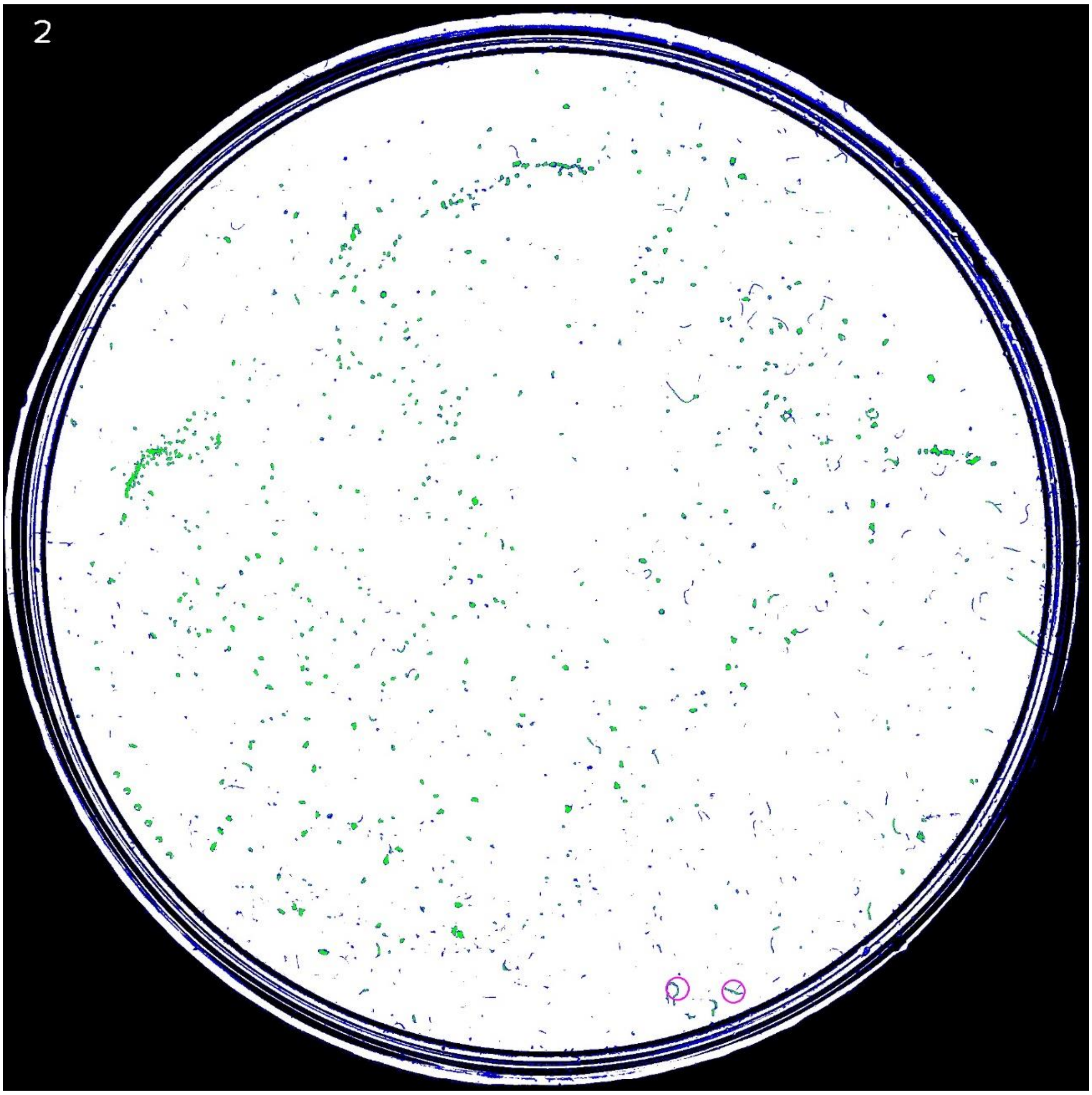

Supplementary Figure S3. Image processing results in a dirt scenario. In this case, two live worms were detected. Each living worm is shown into a pink circumference.

Manual and automated lifespan comparison of daf-2 mutant strain.

This experiment was performed to analyse the post-processing filter when using different strains. The post-processing filter has no effect on the curves if they do not present any error cases. Therefore, an experiment with some errors was required to analyse the effect of the post-processing filter.

This experiment presented some additional occluded zones, such as shown in figure S3, which increased the probability of errors during the entire assay.

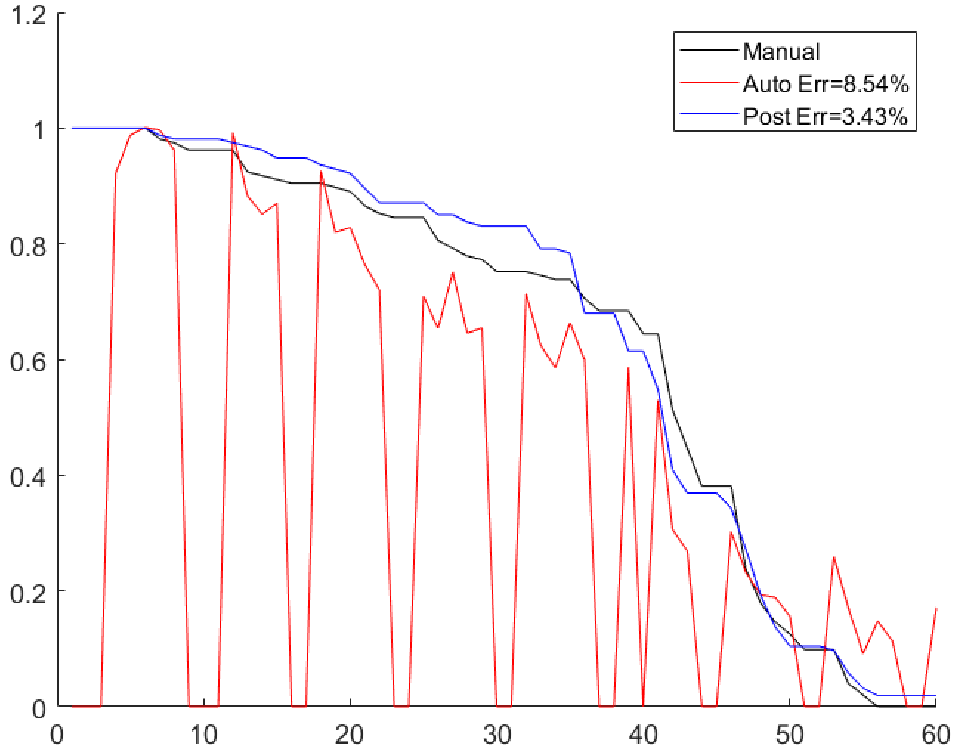

Supplementary Figure S4. Manual and automated lifespan comparison of daf-2 mutant strain. The graph obtained with Matlab R2020b. The automated curve obtained with SIVIS is shown in red. Post process of correction algorithms enabled correct survival measurement of lifespan in long-lived strains like CB1370 (daf-2).
